# Sacubitril/Valsartan Alleviates Cardiac Remodeling and Dysfunction in L-NAME-Induced Hypertension and Hypertensive Heart Disease

**DOI:** 10.3390/biomedicines12040733

**Published:** 2024-03-25

**Authors:** Peter Stanko, Kristina Repova, Tomas Baka, Kristina Krajcirovicova, Silvia Aziriova, Andrej Barta, Stefan Zorad, Michaela Adamcova, Fedor Simko

**Affiliations:** 1 Institute of Pathophysiology, Faculty of Medicine, Comenius University, 81108 Bratislava, Slovakia; pete.stanko@gmail.com (P.S.); kristina.repova@fmed.uniba.sk (K.R.); tomasko.baka@gmail.com (T.B.); krikratina@gmail.com (K.K.); silvia.aziriova@gmail.com (S.A.); 2 Department of Pneumology, Phthisiology and Functional Diagnostics, Slovak Medical University and Bratislava University Hospital, 82606 Bratislava, Slovakia; 3 Institute of Normal and Pathological Physiology, Centre of Experimental Medicine, Slovak Academy of Sciences, 81371 Bratislava, Slovakia; andrej.barta@savba.sk; 4 Institute of Experimental Endocrinology, Biomedical Research Center, Slovak Academy of Sciences, 84505 Bratislava, Slovakia; stefan.zorad@savba.sk; 5 Department of Physiology, Faculty of Medicine in Hradec Kralove, Charles University, 50003 Hradec Kralove, Czech Republic; adamcova@lfhk.cuni.cz; 6 3rd Department of Internal Medicine, Faculty of Medicine, Comenius University, 83305 Bratislava, Slovakia

**Keywords:** sacubitril/valsartan, L-NAME, hypertensive heart disease, hypertrophy, fibrosis, prolactin

## Abstract

There is ample evidence on the benefit of angiotensin receptor-neprilysin inhibitors (ARNIs) in heart failure, yet data regarding the potential protective action of ARNIs in hypertensive heart disease are sparse. The aim of this study was to show whether an ARNI exerts a protective effect in a model of Nω-nitro-L-arginine methyl ester (L-NAME)-induced hypertension with a hypertensive heart and to compare this potential benefit with an angiotensin-converting enzyme inhibitor, captopril. Five groups of adult male *Wistar rats* were studied (14 per group) for four weeks: untreated controls; ARNI (68 mg/kg/day); L-NAME (40 mg/kg/day); L-NAME treated with ARNI; and L-NAME treated with captopril (100 mg/kg/day). L-NAME administration induced hypertension, accompanied by increased left ventricular (LV) weight and fibrotic rebuilding of the LV in terms of increased concentration and content of hydroxyproline in insoluble collagen and in total collagen and with a histological finding of fibrosis. These alterations were associated with a compromised systolic and diastolic LV function. Treatment with either an ARNI or captopril reduced systolic blood pressure (SBP), alleviated LV hypertrophy and fibrosis, and prevented the development of both systolic and diastolic LV dysfunction. Moreover, the serum levels of prolactin and prolactin receptor were reduced significantly by ARNI and slightly by captopril. In conclusion, in L-NAME-induced hypertension, the dual inhibition of neprilysin and AT1 receptors by ARNI reduced SBP and prevented the development of LV hypertrophy, fibrosis, and systolic and diastolic dysfunction. These data suggest that ARNI could provide protection against LV structural remodeling and functional disorders in hypertensive heart disease.

## 1. Introduction

Arterial hypertension is the principal risk factor for cardiovascular diseases [1] resulting in target organ damage, including heart, kidney, and brain damage. Hypertensive heart disease, due to chronic pressure overload, is characterized by structural remodeling of the LV in terms of hypertrophic growth and fibrosis, which are associated with a worsening of diastolic and systolic function, alterations in energetic metabolism, and electrical abnormalities [2]. Although initial mass enlargement aims to normalize LV wall stress under conditions of higher hemodynamic load while maintaining adequate performance, thus representing a compensatory reaction, maladaptive growth of the hypertensive heart in later periods is associated with worse prognosis and frequently results in the development of overt heart failure (HF). Thus, LV hypertrophy has been a target of therapeutic interventions aimed at mitigating or even reversing pathological heart growth, and novel means of treatment are continuously being searched for [3].

In the population with HF, the inhibition of neurohumoral activation by angiotensin inhibitors, beta-blockers, and aldosterone receptor blockers is an effective means of reducing mortality. Besides the inhibition of excessive activation of heart performance and vasoconstriction, the promotion of hormones with vasodilative and antiproliferative action is considered for HF treatment. Natriuretic peptides (NPs), which are released during atrial and ventricular dilation due to pressure or volume overload [4], have been shown to exert desirable protective effects [5,6]. In the PARADIGM-HF trial, a significant reduction of cardiovascular events and mortality in patients with systolic HF was achieved by increasing plasma NP levels via inhibition of the endopeptidase neprilysin by sacubitril in combination with the angiotensin II receptor blocker valsartan [7]. However, the PARAGON-HF (Prospective Comparison of ARNI (angiotensin receptor-neprilysin inhibitor) with ARB (angiotensin-receptor blockers) Global Outcomes in HF with Preserved Ejection Fraction) trial has not shown any benefit of sacubitril–valsartan in patients who had HF with preserved ejection fraction [8]. According to the latest ACC/AHA/HFSA Guideline for the Management of Heart Failure, ARNI is recommended to reduce mortality and morbidity in patients with HF with reduced ejection fraction (HFrEF) and NYHA class II to III [9].

In contrast to the accepted evidence of the mortality-reducing benefit of ARNI in HF, there is a shortage of data regarding the potential protective action of ARNI on the hypertensive heart in patients with increased blood pressure [10,11]. Considering that hypertensive heart disease is a relatively common pathology, the possible protective effect of ARNI in this indication could bring considerable benefits to the population. The aim of our study was to detect the potential cardioprotective effects of ARNI in a model of L-NAME-induced hypertension with a hypertensive heart and to compare it with an angiotensin-converting enzyme inhibitor, captopril.

## 2. Materials and Methods

### 2.1. Animals and Treatment

Adult (12-week-old) male *Wistar rats* (Department of Toxicology and Laboratory Animals Breeding, Slovak Academy of Sciences, Dobra Voda, Slovakia) were randomly divided into five groups (14 per group) with the following treatment lasting for four weeks: no treatment (C); treated with ARNI (68 mg/kg/day; Novartis, Basel, Switzerland) (ARNI); treated with L-NAME (40 mg/kg/day; Cayman Chemical Company, Ann Arbor, MI, USA) (LN); LN treated with ARNI (LN + ARNI); and LN treated with captopril (100 mg/kg/day; Cayman Chemical Company, Ann Arbor, MI, USA) (LN + CAP). The medications were dissolved in drinking water and aligned with daily water consumption. The rats were kept in separate cages and were given an unlimited supply of a regular pellet diet. They were kept in standard laboratory conditions, including a 12 h light and dark cycle, a temperature of 22 ± 2 °C, and humidity of 55 ± 10%. The study was conducted in compliance with the Guide for the Care and Use of Laboratory Animals published by the US National Institutes of Health (NIH publication no. 85-23, revised 1996). The Ethics Committee of the Institute of Pathophysiology, Faculty of Medicine, Comenius University, Bratislava, Slovakia approved the protocol (approval number: 809/19-221/3; approval date: 23 April 2019).

### 2.2. Blood Pressure Measurement, Collection and Processing of Samples

During treatment, the SBP was measured once a week using a non-invasive tail-cuff plethysmography (Hugo Sachs Elektronik, Freiburg, Germany). Following the four-week treatment period, the rats were humanely euthanized by isoflurane inhalation. Body weight (BW), tibia length (TL), left ventricular weight (LVW), right ventricular weight (RVW), and lungs and liver weights were measured. Afterward, the left ventricular weight to body weight (LVW/BW), left ventricular weight to tibia length (LVW/TL), right ventricular weight to body weight (RVW/BW), and right ventricular weight to tibia length (RVW/TL) ratios were calculated. LV tissue samples were frozen at −80 °C and subsequently analyzed for hydroxyproline concentrations.

### 2.3. Echocardiography

All animals underwent transthoracic echocardiography after four weeks of treatment using a 14 MHz matrix probe (M12L) and the GE Medical Vivid 7 Dimension System (GE Medical Systems CZ Ltd., Prague, Czech Republic) [12]. The animals were anesthetized throughout the procedure with isoflurane (2.5% inspiratory concentration at a flow rate of 2 L/min) while breathing spontaneously. In this study, the thoracic wall of the rats was shaved while they were placed in a supine position on a warming pad at a temperature of 38 °C. Continuous monitoring of the rats’ heart rate (HR) and body temperature was carried out during the procedure.

Using the parasternal long-axis images, the end-diastolic (IVSd) and end-systolic thickness of the interventricular septum (IVSs) were measured. The LV end-diastolic (LVIDd) and end-systolic (LVIDs) internal diameters, the LV end-diastolic (PWd) and end-systolic (PWs) posterior wall thickness, and end-diastolic (EDV) and end-systolic volumes (ESV) were measured using anatomical M-mode in a parasternal long-axis view.

The systolic function of the LV was evaluated by calculating the LV fractional shortening (LVFS) and ejection fraction (LVEF) using the Teichholz formula. The diastolic function of the LV was evaluated by measuring the diastolic transmitral peak early (E) and late (A) filling velocities from the two-dimensionally guided Doppler spectra of mitral inflow in the apical four-chamber view. The E/A ratio and deceleration time (DecT) were then calculated to evaluate the LV diastolic function.

From the pulsed-wave Doppler flow in the apical five-chamber view, the LV inflow (mitral inflow) and outflow (aortic flow velocity) signals were recorded, and the isovolumic relaxation time (IVRT), the time between the closing of the aortic valve and the opening of the mitral valve, was calculated.

An experienced echocardiographer blinded to the group identity performed all echocardiography measurements, which were averaged over three consecutive cardiac cycles.

### 2.4. Hydroxyproline in the Left Ventricle

To isolate collagenous proteins in the LV, LV samples were treated with different buffers [13]. Soluble collagenous proteins were extracted using CH3COOH-pepsin buffer (pH 1.4, 24 h at 4 °C), and the remaining insoluble collagenous proteins were extracted using NaOH (1.1 mol/L, 45 min at 105 °C). Subsequently, the hydrolyzed samples were oxidized using chloramine T and acetate-citrate buffer at pH 6.0. After incubation (20 min at room temperature), the reaction was stopped by adding Ehrlich’s reagent, followed by incubation at 65 °C for 15 min. Spectrophotometry at 550 nm was used to measure the LV hydroxyproline concentration, a marker of LV fibrosis, in both collagenous fractions. The hydroxyproline content in the LV was then calculated and expressed as mg per total weight of the LV.

### 2.5. Quantitative Analysis of the Left Ventricular Fibrosis

The LV samples from 6 subjects per treatment group, preserved in a solution of 4% formaldehyde, underwent embedding in paraffin and were subsequently sectioned into slices measuring 5 μm in thickness. Following this, sample sections underwent a process of deparaffinization, rehydration, and staining with picrosirius red (PSR) for a quantitative assessment of LV fibrosis. Photomicrographs were captured utilizing a NIKON Eclipse Ti C2+ microscope (NIKON, Tokyo, Japan) equipped with polarized light, and thereafter subjected to analysis using NIKON NIS-Elements Analysis software (v 4.50.00, NIKON, Tokyo, Japan) and ImageJ software (1.54f, National Institutes of Health, Bethesda, MD, USA) [14].

To achieve a quantitative assessment of LV fibrosis, sections stained with PSR were subjected to analysis at a magnification of 10× using polarized light microscopy in conjunction with ImageJ software (1.54f). PSR enhances the birefringence of collagen fibers in a manner dependent on their type, thereby facilitating the visualization of thick type I collagen (Col I, 1.6–2.4 μm in diameter) appearing in red–orange hues and thin type III collagen (Col III, <0.8 μm in diameter) appearing in green–yellow hues. By appropriately configuring the hue, brightness, and saturation thresholds of the color spectrum, the areas exhibiting red–orange and green–yellow shades were quantified as percentages of the total area of interest (AOI) through ImageJ processing. Upon ascertaining the volume of Col I and Col III, we proceeded to compute the sum of Col I and Col III (Col I + III), as well as the ratio of Col I to Col III (Col I/III).

### 2.6. Serum Concentration of Prolactin and Prolactin Receptor

Rat enzyme-linked immunosorbent assay (ELISA) kits designed for the quantification of prolactin and prolactin receptor (prolactin R) were employed to determine their respective serum concentrations in 6 subjects per treatment group (RayBiotech Life, Inc., 3607 Parkway Ln, Suite 200, Peachtree Corners, GA, USA).

### 2.7. Statistical Analysis

The results are presented as the means and standard error of the mean (SEM). To analyze the data statistically, we used a one-way, repeated-measures analysis of variance (ANOVA) followed by multiple comparisons with a Bonferroni post hoc test. We considered differences to be significant if the p-value was less than 0.05. All statistical analyses were conducted using GraphPad Prism 9 for Windows (GraphPad Software, La Jolla, CA, USA).

## 3. Results

### 3.1. Cardiovascular Parameters

#### 3.1.1. Systolic Blood Pressure Was Reduced by ARNI and Captopril in L-NAME-Induced Hypertension

**Figure 1:**
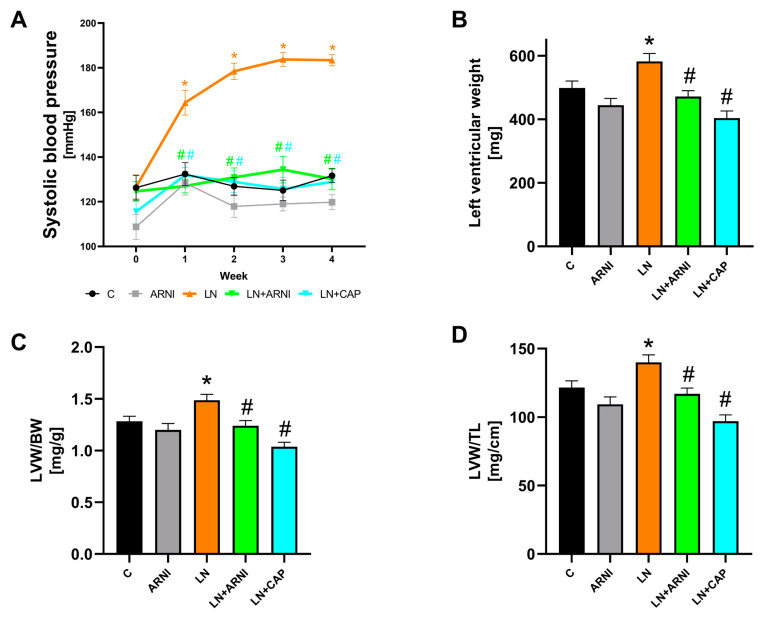
Effect of ARNI and captopril on systolic blood pressure (**A**), left ventricular weight (**B**), left ventricular weight to body weight (LVW/BW) (**C**), and to tibia length ratio (LVW/TL) (**D**) in L-NAME-induced hypertension and hypertensive heart disease after four weeks of treatment. C, controls; LN, L-NAME; ARNI, sacubitril/valsartan; CAP, captopril. * *p* < 0.05 vs. C; # *p* < 0.05 vs. LN.

After four weeks of treatment, the SBP was 131.7 ± 3.02 mmHg in the controls, while ARNI administration did not change it (Figure 1A). The chronic L-NAME treatment significantly increased the SBP to 183.4 ± 2.52 mmHg (by 39%, *p* < 0.05), while both ARNI (by 29%, *p* < 0.05) and captopril (by 30%, *p* < 0.05) significantly reduced it.

#### 3.1.2. ARNI and Captopril Reduced LVW in L-NAME-Induced Hypertension

Following a period of 4 weeks of treatment, discernible alterations in body weight were not observed across the respective groups (Table 1). The absolute LV weight in the control group was 499 ± 21.81 mg, while ARNI treatment did not change it (Figure 1B). The L-NAME administration increased LV weight to 582 ± 25 mg (by 17%, *p* < 0.05), while both ARNI (by 24%, *p* < 0.05) and captopril (by 18%, *p* < 0.05) reduced it significantly. In the control group, the LVW/BW and LVW/TL ratio after four weeks was 1.28 ± 0.05 mg/g and 135.1 ± 5 mg/cm, respectively, while ARNI administration did not change it (Figure 1C,D). The L-NAME treatment increased the LVW/BW and LVW/TL ratio significantly to 1.49 ± 0.06 mg/g (by 16%, *p* < 0.05) and 140.12 ± 5.3 mg/cm (15%, *p* < 0.05), respectively, and ARNI (by 17% and 16%, respectively, *p* < 0.05) and captopril (by 30% and 31%, respectively, *p* < 0.05) reduced it significantly. The BW, LVW, RVW, RVW/BW, and RVW/TL ratios are described in Table 1.

#### 3.1.3. ARNI and Captopril Improved LV Function in L-NAME-Induced Hypertension

**Figure 2:**
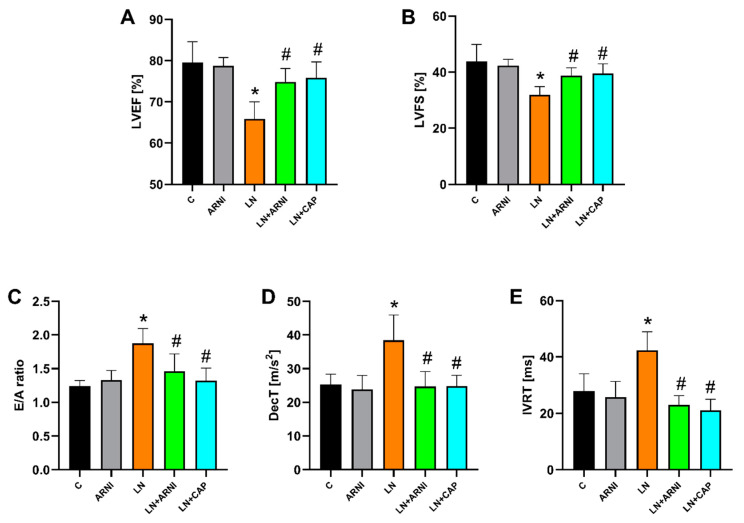
Effect of ARNI and captopril on left ventricular ejection fraction (LVEF) (**A**), left ventricular fractional shortening (LVFS) (**B**), the ratio of the diastolic transmitral peak early and late filling velocities (E/A ratio) (**C**), deceleration time (DecT) (**D**) and isovolumic relaxation time (IVRT) (**E**) in L-NAME-induced hypertension and hypertensive heart disease after four weeks of treatment. C, controls; LN, L-NAME; ARNI, sacubitril/valsartan; CAP, captopril. * *p* < 0.05 vs. C; # *p* < 0.05 vs. LN.

After four weeks, the echocardiographic parameters of the control group did not change with ARNI treatment. However, administration of L-NAME decreased LVEF by 17% (*p* < 0.05) and LVFS by 27% (*p* < 0.05) (Figure 2A,B). On the other hand, both ARNI and captopril significantly increased these parameters in the L-NAME-administered group. In the hypertensive L-NAME group, ARNI increased LVEF by 14% (*p* < 0.05) and LVFS by 21% (*p* < 0.05), while captopril increased LVEF by 15% (*p* < 0.05) and LVFS by 24% (*p* < 0.05).

In contrast, LV diastolic function parameters, such as the E/A ratio (by 51%, *p* < 0.05), DecT (by 52%, *p* < 0.05), and IVRT (by 52%, *p* < 0.05), increased after L-NAME administration (Figure 2C–E). Both ARNI and captopril effectively reduced these parameters in the L-NAME group, as follows: ARNI reduced the E/A ratio by 22% (*p* < 0.05), DecT by 36% (*p* < 0.05), and IVRT by 46% (*p* < 0.05), while captopril reduced the E/A ratio by 29% (*p* < 0.05), DecT by 36% (*p* < 0.05) and IVRT by 50% (*p* < 0.05).

The HR, IVSd, IVSs, LVIDd, LVIDs, PWd, PWs, EDV, and ESV are described in Table 2.

### 3.2. Hydroxyproline Concentration and Content in Soluble, Insoluble and Total Collagenous Proteins in the Left Ventricle Was Decreased by ARNI and Captopril in L-NAME-Induced Hypertension

**Figure 3:**
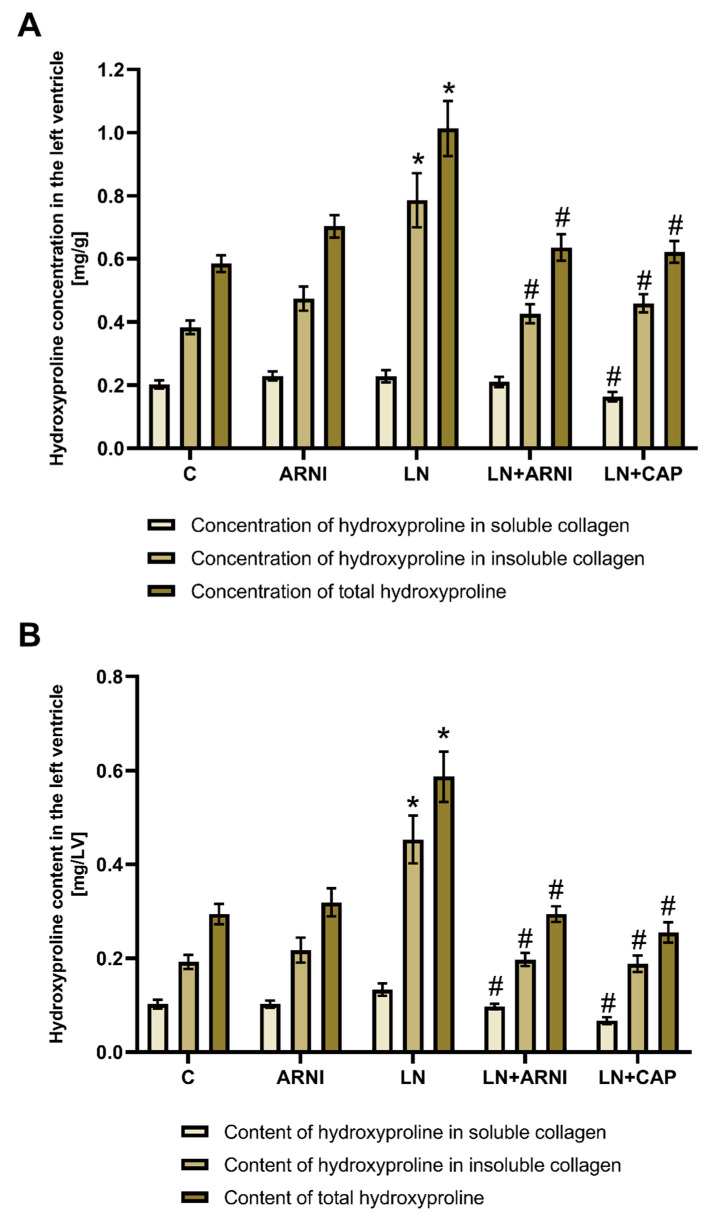
Effect of ARNI and captopril on the concentration (**A**) and content (**B**) of hydroxyproline in soluble and insoluble collagenous proteins and total hydroxyproline in L-NAME-induced hypertension and hypertensive heart disease after four weeks of treatment. C, controls; LN, L-NAME; ARNI, sacubitril/valsartan; CAP, captopril. * *p* < 0.05 vs. C; # *p* < 0.05 vs. LN.

After four weeks of treatment, the hydroxyproline concentration in the soluble collagenous protein was 0.202 ± 0.013 mg/g in the controls, and ARNI did not change it (Figure 3A). The L-NAME administration increased the hydroxyproline concentration in the soluble collagenous protein numerically to 0.288 ± 0.019 mg/g (ns), while captopril only significantly reduced it, by 28% (*p* < 0.05). In the controls, the hydroxyproline concentration in the insoluble collagenous fraction was 0.384 ± 0.022 mg/g, while ARNI had no effect. After four weeks of L-NAME treatment, the concentration of hydroxyproline in the insoluble collagenous fraction was 0.786 ± 0.085 mg/g, and both ARNI (by 46%, *p* < 0.05) and captopril (by 42%, *p* < 0.05) reduced it significantly. The total hydroxyproline concentration was 0.585 ± 0.026 mg/g in the controls, while ARNI had no effect. L-NAME administration increased the total hydroxyproline concentration to 1.014 ± 0.087 mg/g (*p* < 0.05), and both ARNI (by 37%, *p* < 0.05) and captopril (by 39%, *p* < 0.05) reduced it significantly.

After four weeks of treatment, the hydroxyproline content in the soluble collagenous proteins was 0.102 ± 0.009 mg/LV in the controls, and ARNI did not change it (Figure 3B). The L-NAME administration increased the content in the soluble collagenous proteins numerically to 0.133 ± 0.014 mg/LV (ns), and both ARNI (by 27%, *p* < 0.05) and captopril (by 50%, *p* < 0.05) reduced it significantly. The hydroxyproline content in the insoluble collagenous fraction was 0.192 ± 0.015 mg/LV in the controls, and ARNI had no effect. After L-NAME treatment, the hydroxyproline content in the insoluble collagenous proteins was 0.453 ± 0.051 mg/LV (*p* < 0.05), and both ARNI (by 56%, *p* < 0.05) and captopril (by 59%, *p* < 0.05) reduced it significantly. The total hydroxyproline content was 0.294 ± 0.021 mg/LV in the controls, while ARNI did not change it. L-NAME administration increased the total hydroxyproline concentration to 0.587 ± 0.054 mg/LV (*p* < 0.05), and both ARNI (by 50%, *p* < 0.05) and captopril (by 57%, *p* < 0.05) reduced it significantly.

### 3.3. Left Ventricular Fibrosis Was Reduced by ARNI and Captopril in L-NAME-Induced Hypertension

**Figure 4:**
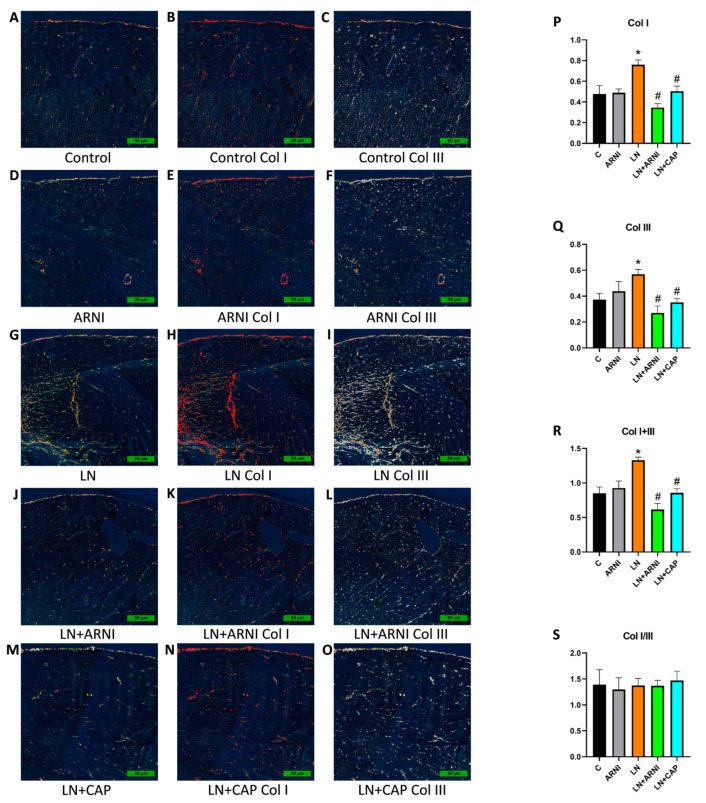
Effect of ARNI and captopril on left ventricular fibrosis in L-NAME-induced hypertension and hypertensive heart disease. PSR-stained sections at 10× magnification using polarized light microscopy showing collagen I in red and collagen III in white (**A**–**O**), the volume of collagen I (Col I) (**P**), collagen III (Col III) (**Q**), the sum of collagen I and III (Col I + III) (**R**), and the ratio of Col I/Col III (Col I/III) (**S**). C, controls; LN, L-NAME; ARNI, sacubitril/valsartan; CAP, captopril. * *p* < 0.05 vs. C; # *p* < 0.05 vs. LN.

Following a duration of four weeks from the initiation of the experiment, the volume of Col I and Col III in the LV AOI in the control group was 0.47 ± 0.2 and 0.37 ± 0.12, respectively, and were not affected by ARNI (Figure 4P,Q); L-NAME increased (*p* < 0.05) the proportion of Col I (by 59%, *p* < 0.05) and Col III (by 52%, *p* < 0.05). In the L-NAME group, both ARNI and captopril decreased (*p* < 0.05) the proportion of Col I (by 54% and 34%, respectively) and Col III (by 53% and 38%, respectively). The sum of Col I + III in the control group was 0.85 ± 0.22 and ARNI did not influence it (Figure 4R); chronic administration of L-NAME increased the sum of Col I and Col III (by 57%, *p* < 0.05), while both ARNI and captopril significantly reduced it (by 54% and 35%, respectively, *p* < 0.05). The Col I/III ratio was 1.4 ± 0.7 in the control group and no statistically significant differences were observed among the groups (Figure 4S).

### 3.4. Serum Levels of Prolactin and Prolactin Receptor Were Reduced by ARNI in L-NAME-Induced Hypertension

**Figure 5:**
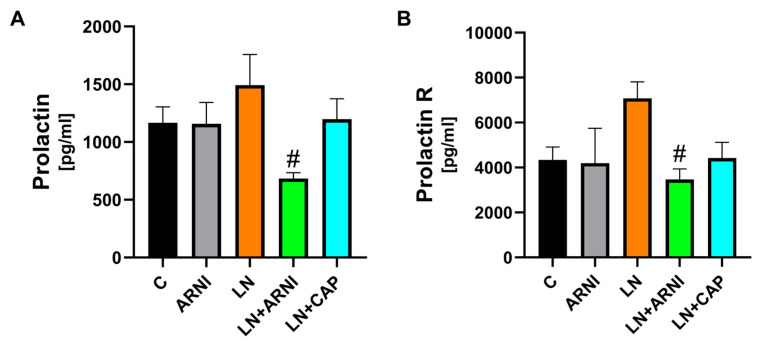
Effect of ARNI and captopril on serum levels of prolactin (**A**) and prolactin receptor (prolactin R) (**B**) in the L-NAME-induced hypertension and hypertensive heart disease. C, controls; LN, L-NAME; ARNI, sacubitril/valsartan; CAP, captopril. # *p* < 0.05 vs. LN.

After four weeks, the serum level of prolactin and prolactin R in the control group was 1 165.1 ± 139.6 pg/mL and 4 336.7 ± 576.8 pg/mL, respectively, and ARNI did not have an effect on these molecules (Figure 5A,B). L-NAME numerically increased serum levels of prolactin (by 28%, *p* < 0.05) and prolactin R (by 63%, *p* < 0.05), while only ARNI reduced these monitored serum parameters (by 54% and 51%, respectively, *p* < 0.05). Captopril reduced prolactin or prolactin receptor levels only numerically (without statistical significance).

## 4. Discussion

In this experiment, four weeks of L-NAME administration resulted in hypertension accompanied by increased LV weight and fibrotic rebuilding of the LV in terms of increased hydroxyproline concentration and content and histological findings of increased fibrosis. These hemodynamic and structural alterations were associated with compromised systolic and diastolic LV function. Treatment with either ARNI or captopril reduced systolic blood pressure, alleviated LV hypertrophy and fibrosis, and prevented the development of LV dysfunction. Additionally, the serum level of prolactin and prolactin receptor, supposedly associated with endothelial dysfunction, pathological remodeling, and compromised cardiovascular health, was significantly reduced by ARNI and slightly by captopril.

L-NAME-induced hypertension is a well-established model of hypertension and target organ damage. L-NAME acts as a false substrate for endothelial nitric oxide synthase (eNOS), thus reducing the formation of nitric oxide (NO), resulting in the development of NO-deficient hypertension [14]. Besides the shortage of vasodilative action of NO itself, reduced NO availability in the renal artery is thought to stimulate renin release, followed by angiotensin II (Ang II)—aldosterone axis activation in the circulation [15] and in tissues such as the heart and aorta [16] or kidney [14,17], thus contributing to hypertension development and end-stage organ damage [15]. Nonetheless, our recent investigation revealed that L-NAME-induced hypertension is linked not to heightened, but rather to the normal or reduced activation of the renin–angiotensin II system (RAS) in both serum and LV tissue [18]. The normal-to-low level of renin–angiotensin system activation has also been previously documented in spontaneously hypertensive rats [19]. Yet, the serum aldosterone level and the ratio of aldosterone to Ang II were increased in L-NAME-hypertension [18,20], which is consistent with the results obtained by other research groups [21,22]. In agreement with our recent results with an L-NAME model of hypertension, aldosterone activation was previously demonstrated in various models of experimental hypertension [23]. Consequently, it is possible that the pivotal factor contributing to LV remodeling in L-NAME-induced hypertension is the activation of aldosterone rather than Ang II [18]. The described pro-proliferative effect of aldosterone receptor stimulation [24] with the enhancement of the collagenous concentration and content and histological signs of fibrosis in myocardial tissue seen in this experiment may underly the deterioration of diastolic and systolic LV function. The rise in the level of insoluble collagen with numerous cross-linking in particular may deteriorate the myocardial elasticity and participate in the LV diastolic dysfunction development [18].

Neprilysin is an endopeptidase cleaving various biologically active peptides. Some of these compounds, such as atrial natriuretic peptide (ANP), brain natriuretic peptide (BNP), and C-type natriuretic peptide (CNP), along with adrenomedullin and bradykinin, support vasodilation, natriuresis, insulin sensitivity, and antifibrotic action [25]. On the other hand, neprilysin also degrades Ang II or endothelin-1 with opposing effects in terms of vasoconstriction, pro-aggregation, and pro-proliferation. Thus, inhibition of neprilysin by sacubitril not only increases the bioavailability of presumably protective substances but also raises the levels of vasoconstrictors and growth promotors, including Ang II. This might partially counterbalance the protective effects of natriuretic peptides [26]. To avoid these adverse effects, neprilysin inhibitor was combined with the Ang II type 1 (AT1) receptor blocker valsartan in one pill (sacubitril/valsartan—ARNI) in order to eliminate the undesirable effect of increased Ang II [27,28].

The neprilysin AT1 blocker ARNI has been shown to be effective in a number of heart failure studies [29] and is recommended for the treatment of HF with reduced ejection fraction [9]. Yet, it is not entirely clear whether ARNI could provide additional benefits in the treatment of hypertension and in the protection of the hypertensive heart [10,11]. The situation with hypertensive heart disease is complex because the nature of essential hypertension and the corresponding target organ damage can be related to various interfering factors, such as endothelial dysfunction, chronic neurohumoral activation, or genetic predisposition. Therefore, testing the effect of a particular drug in different experimental models of hypertension is unavoidable. Although ARNI has been investigated in several models of hypertension and associated cardiac injury, to the best of our knowledge, ARNI has not been used in the L-NAME model of hypertension, which is primarily induced by a deficit of nitric oxide production and is considered to be a NO-deficient model of hypertension [15,30]. The disclosing of the protective effect of ARNI in the L-NAME model in this work may contribute to understanding the pathomechanism of ARNI in hypertensive disease with endothelial dysfunction and limited nitric oxide production and could support the consideration of ARNI in the treatment of essential hypertension, which is currently not the case [10].

In our experiment, both ARNI and the angiotensin-converting enzyme (ACE)-inhibitor captopril reduced SBP and LV hypertrophy, along with improving the systolic and diastolic functional parameters of the LV. These results are in agreement with previous results from our and other laboratories. In L-NAME hypertension, captopril prevented SBP enhancement and the development of LV hypertrophy and fibrosis [30] and reduced DNA and RNA concentration and protein synthesis in the LV, aorta, kidney, and brain [31] without restoring nitric oxide synthase activity and even induced the regression of hypertrophic and fibrotic remodeling, when LV hypertrophy was already developed [32]. Although ARNI was not investigated in L-NAME hypertension, protection was shown in various other models of experimental cardiovascular pathologies. ARNI treatment attenuated structural changes in terms of myocardial hypertrophy and fibrosis after myocardial infarction [33,34] and in salt-induced heart damage in rats [35], and reduced cardiomyocyte hypertrophy in Ang II-induced hypertension [36]. ARNI improved LV function in anthracycline-induced cardiomyopathy [37] in models of myocardial infarction in rats [33] and mice [34], as well as in high-salt diet-induced diastolic dysfunction in rats [35]. In SHR, ARNI improved atrial remodeling and reduced susceptibility to atrial fibrillation [38], and a novel ARNI S086 effectively reduced myocardial cell necrosis and fibrosis development [39]. A meta-analysis of clinical studies spanning the decade from 2010 to 2019 [40] demonstrated the remarkable ability of ARNI to reverse LV remodeling in individuals suffering from HF accompanied by systolic dysfunction [40], and ARNI’s antifibrotic potential is to be investigated in the REVERSE-LVH clinical trial in a regression experiment on hypertensive patients with LVH [11].

Various cardiac markers in plasma are under investigation to obtain a general overview of the extent of cardiovascular changes induced by hypertension in clinical or experimental conditions. One of them, prolactin, a polypeptide hormone of lactation, exerts pleiotropic cytokine effects that are associated with hypertension and cardiac remodeling [41]. Elevated serum prolactin has been associated with the development of hypertension through a reduction in endothelial nitric oxide synthase activity in mice [42]. In addition, hyperprolactinemia is associated with endothelial dysfunction and impaired insulin sensitivity [43,44], increased arterial stiffness [45], and the risk of atherosclerosis and cardiovascular events in high-risk patients [44]. In addition, prolactin receptor expression appears to be linked to myocardial hypertrophy [46]. Although prolactin did not reliably reflect cardiometabolic risk in an analysis of 3232 subjects from the Framingham Heart Study [47], a recent study of 10,907 patients with type 2 diabetes showed a positive association of serum prolactin with mortality [48], and a recent meta-analysis of 14 studies involving 23,596 patients confirmed that serum prolactin is an independent predictor of all-cause mortality and cardiovascular mortality [49]. The results of our experiment correlated well with these data from the literature. An increase in prolactin and prolactin receptor levels was revealed in the L-NAME group, which is consistent with the LV remodeling observed in this experiment and the decreased activity of endothelial NO activity in the heart, aorta, kidneys, and brain mentioned in a number of our previous studies [30,31,50,51]. Importantly, ARNI significantly and captopril slightly reduced serum prolactin and prolactin R levels, suggesting slightly better protective potential with ARNI, despite similar hemodynamic and structural effects of the two drugs.

The findings of our current experiment support the hypothesis that the simultaneous inhibition of neprilysin and AT1 receptors could exert protective effects in the hypertensive heart. These benefits include not only mitigating structural remodeling but also averting functional impairments. It has to be recognized, however, that in most animal and clinical experiments, ARNI outperformed the ACE-inhibition or AT1 blockade, whereas in our experiment with an L-NAME-induced hypertensive heart, ARNI and captopril yielded comparable outcomes. The appropriate dose of ARNI and captopril should thus be considered. Both the dual inhibitor and the classical ACE-inhibitor captopril were given in a high pharmacologic dose. A dose of captopril of 100 mg/kg was chosen in relation to a number of our prior experiments, where this dose yielded protection while keeping side effects unnoticeable. The dose of ARNI of 68 mg/kg was in agreement with the highest dose used in experiments from other laboratories. The adequacy of the choice of both doses is supported by the equal SBP reduction by both drugs during the whole experiment. However, one should consider several other issues, which may have potentially influenced the level of protection. First, captopril (unlike enalapril used in the PARADIGH-HF Trial) contains SH groups that are claimed to enhance its antioxidant properties [52]. In the condition of decreased NO production in the L-NAME-induced hypertension, this factor might have improved NO availability and thus enhanced the protection by captopril. Second, L-NAME-induced hypertension was previously shown to be a model with low renin–angiotensin II pathway activation. Under these circumstances, the space for RAS inhibitors might be restricted, thus hiding subtle differences of the drugs tested. Third, in a condition of hypertension with the absence of HF and fluid accumulation, there may be no need for increased natriuretic peptide availability. Furthermore, even the very similar effects of ARNI and captopril on the surrogate endpoint represented by hypertensive heart diseases do not automatically indicate the same effect on morbidity and mortality in subjects with hypertensive heart remodeling, which could only be disclosed in a long-term experiment focused on the hard endpoints. This consideration is supported by the more pronounced attenuation of the serum level of prolactin and prolactin R by ARNI than by captopril.

In conclusion, we have shown that in L-NAME-induced hypertension, the dual inhibition of neprilysin and AT1 receptor by ARNI reduced SBP and prevented the development of LV hypertrophy, fibrosis, and systolic and diastolic dysfunction. The data suggest that in hypertensive individuals with hypertensive heart disease, ARNI could yield protection against LV structural remodeling and functional disorders.

## 5. Limitations

Investigating natriuretic peptides could help reflect neurohumoral modifications. However, in a number of papers on ARNI from several laboratories, NPs were not presented as standard. This may be due to the following reasons. Since the recent advent of ARNIs, it is necessary to investigate how these two biomarkers should be interpreted in HF or ventricular dysfunction. ARNI use is associated with a decrease in NT-proBNP but an increase in BNP levels [53]. Moreover, due to the large species specificities of NP, it is necessary to use rat-specific kits, while standardization in experimental animals is lacking. Different kits have been used with different results in the literature. For these reasons, it seems that only experienced laboratories are able to adequately interpret complex changes in NPs under specific conditions.

Furthermore, in vitro experiments of the effects of neurohumoral inhibitors on isolated myocardial cells, along with thorough histological investigation, may supply deeper insight into the protective effects of the drug used. However, these investigations were beyond the technical equipment and possibilities of our laboratory.

## Figures and Tables

**Table 1:** Body weight (BW), tibia length (TL), right ventricular weight (RVW), RVW to body weight (BW) ratio (RVW/BW), RVW to TL ratio (RVW/TL), lungs weight, and liver weight in L-NAME-induced hypertension and hypertensive heart disease after four weeks of treatment. Values are mean ± SEM. C, controls; LN, L-NAME; ARNI, sacubitril/valsartan; CAP, captopril.

	C	ARNI	LN	LN + ARNI	LN + CAP
BW [mg]	388 ± 5.5	370 ± 5.6	391 ± 7.94	380 ± 9.23	388 ± 11.43
TL [cm]	4.09 ± 0.04	4.06 ± 0.04	4.14 ± 0.03	4.02 ± 0.07	4.14 ± 0.05
RVW [mg]	172.6 ± 6.90	175.3 ± 5.49	176.00 ± 11.28	167.90 ± 13.29	158.30 ± 7.99
RVW/BW [mg/g]	0.45 ± 0.02	0.47 ± 0.01	0.45 ± 0.03	0.44 ± 0.03	0.41 ± 0.01
RVW/TL [mg/cm]	42.41 ± 2.01	43.57 ± 1.59	42.43 ± 2.59	42.39 ± 3.13	37.62 ± 1.69
Lungs [mg]	1.61 ± 0.05	1.58 ± 0.04	1.53 ± 0.03	1.51 ± 0.03	1.57 ± 0.03
Liver [mg]	11.64 ± 0.27	10.99 ± 0.27	11.89 ± 0.31	11.33 ± 0.4	11.69 ± 0.38

**Table 2:** Effect of ARNI and captopril on left ventricular echocardiographic functional parameters in L-NAME-induced hypertension and hypertensive heart disease after four weeks of treatment; heart rate (HR), end-diastolic interventricular septum thickness (IVSd), end-systolic interventricular septum thickness (IVSs), end-diastolic LV internal diameter (LVIDd), end-systolic LV internal diameter (LVIDs), end-diastolic posterior wall diameter (PWd), end-systolic posterior wall diameter (PWs), end-diastolic LV volume (EDV), end-systolic LV volume (ESV). C, controls; LN, L-NAME; ARNI, sacubitril/valsartan; CAP, captopril. * *p* < 0.05 vs. C; # *p* < 0.05 vs. LN.

	C	ARNI	LN	LN + ARNI	LN + CAP
HR [min^−1^]	380.90 ± 0.16	361.43 ± 11.15	343.05 ± 10.17	353.64 ± 11.54	355.55 ± 11.79
IVSd [cm]	0.91 ± 0.02	0.89 ± 0.02	0.94 ± 0.03	0.79 ± 0.02 #	0.73 ± 0.02 #
IVSs [cm]	0.87 ± 0.03	0.85 ± 0.03	0.85 ± 0.03	0.75 ± 0.02 #	0.74 ± 0.03 #
LVIDd [cm]	6.67 ± 0.15	6.55 ± 0.11	6.63 ± 0.16	6.33 ± 0.15	6.20 ± 0.18
LVIDs [cm]	3.77 ± 0.16	3.77 ± 0.08	4.52 ± 0.14 *	3.89 ± 0.12 #	3.75 ± 0.13 #
PWd [cm]	0.98 ± 0.04	0.92 ± 0.02	1.02 ± 0.03	0.83 ± 0.04 #	0.77 ± 0.03 #
PWs [cm]	1.14 ± 0.04	1.05 ± 0.02	1.13 ± 0.03	0.96 ± 0.03 #	0.85 ± 0.03 #
EDV [mL]	0.69 ± 0.04	0.65 ± 0.03	0.68 ± 0.04	0.60 ± 0.04	0.57 ± 0.05
ESV [mL]	0.15 ± 0.01	0.14 ± 0.01	0.24 ± 0.02 *	0.15 ± 0.01 #	0.14 ± 0.01 #

## Data Availability

Data supporting the reported results are available from the corresponding author per request.
